# Joint aspiration and serum markers - do they matter in the diagnosis of native shoulder sepsis? A systematic review

**DOI:** 10.1186/s12891-022-05385-8

**Published:** 2022-05-19

**Authors:** Luis M. Salazar, Jose M. Gutierrez-Naranjo, Clarissa Meza, Andrew Gabig, Aaron J. Bois, Christina I. Brady, Anil K. Dutta

**Affiliations:** 1 UT Health San Antonio, Long School of Medicine, San Antonio, TX USA; 2 UT Health San Antonio, Department of Orthopaedics, Floyd Curl Dr, MC 7774, San Antonio, TX 78229-3900 USA; 3 University of Calgary, Sport Medicine Centre, Calgary, AB Canada

**Keywords:** Shoulder sepsis, Septic arthritis, Glenohumeral joint sepsis, Diagnosis, Shoulder

## Abstract

**Background:**

Septic arthritis of the native shoulder is traditionally diagnosed with the same strategies as knee or hip septic arthritis. However, septic arthritis of the shoulder is frequently a missed or delayed diagnosis. Reliance on aspiration and serum markers has been called into question recently. The purpose of this study was to conduct a systematic review investigating the value of joint aspiration and serum markers in the diagnosis of native shoulder joint sepsis.

**Methods:**

PubMed/MEDLINE, Scopus, and the Cochrane Library were used in the systematic literature search from January 1, 1960, through January 23, 2021. The primary outcome was to report on the synovial white cell count of patients with native shoulder sepsis. Descriptive statistics using percentages, means, and intraclass correlation coefficient (ICC) values were used to summarize the results.

**Results:**

Thirty-one studies, including 25 case series, one case-control, and five cohort studies with a total of 7434 native shoulder joints, were included. There was no standardized approach to diagnosing septic arthritis of the shoulder. Only 10 studies (32%) reported on synovial white cell count with the majority yielding aspiration counts greater than 50,000 cells/mm^3^, although one study was as low as 30,000 cells/mm^3^.

**Conclusions:**

The diagnosis of native shoulder joint sepsis lacks uniformity. Methods used to evaluate shoulder sepsis are heterogeneous and may lead to delays or misdiagnosis with devastating sequelae. Synovial white cell count is underutilized and may also present with a lower value than expected, which is likely related to the time interval between symptom onset and diagnosis.

## Background

Septic arthritis of the shoulder is a less common condition when compared with knee or hip sepsis with potentially devastating sequelae. Accounting for 3 to 15% of all septic arthritis cases, shoulder sepsis can lead to bone and cartilage destruction, osteonecrosis, ankylosis, and even death [1–5]. Persistent shoulder pain and limited range of motion are also common, especially with delays in diagnosis [1, 6]. Shoulder sepsis commonly occurs in patients with medical comorbidities and has a particularly poor prognosis in the immunocompromised and patients with rheumatoid arthritis [7–9]. Studies have also shown that delays in diagnosis consistently produce longer hospital stays and worse functional outcomes [3, 10]. Therefore, timely diagnosis and treatment of septic arthritis of the shoulder is paramount but remains a challenge even for experienced surgeons.

Shoulder sepsis is often misdiagnosed as bursitis, tendinitis, and frozen shoulder, as the most common symptoms include shoulder pain and limited range of motion [5, 6]. Furthermore, traditional methods of evaluating septic arthritis such as analysis of the joint aspirate (cell count/differential and fluid culture), and blood cultures are often unreliable when assessing for septic arthritis of the shoulder. Negative synovial fluid culture results have been reported as high as 47%, and blood cultures only have a 50% positivity rate [1, 11, 12]. Even if these clinical and laboratory findings support the diagnosis of sepsis, they do not reflect the severity of disease, leading to potential undertreatment of patients [13]. Plain radiographs are insensitive, nonspecific, and can miss osteomyelitis, especially during the early stages of septic arthritis [13, 14]. Ultrasonography can detect effusions and synovial changes though osseous changes are difficult to identify [15]. Magnetic resonance imaging (MRI) is becoming an integral part of the diagnostic workup of shoulder sepsis as it is non-invasive and can be used preoperatively to classify the severity of shoulder sepsis and guide the surgical approach for optimal management [14] (Fig. 2a and b).

Debate continues surrounding the ideal treatment of shoulder sepsis though this generally involves an arthroscopic and/or open approach [16–19]. To date, most of the literature has focused on management strategies of shoulder sepsis. However, given the uniqueness and complexity of the presentation of septic arthritis of the shoulder, substantial variability exists in the literature regarding accurate diagnosis, and there is currently no standardized and accepted method. To the best of our knowledge, no systematic review has thoroughly analysed the clinical utility of joint aspiration results used to evaluate and diagnose septic arthritis of the native shoulder joint. Therefore, the purpose of the present systematic review was to methodologically review the value of the synovial white cell count in the setting of native shoulder joint sepsis. The secondary objective of the study was to assess the utility of serum laboratory markers used to assess joint sepsis (e.g., white blood cell count, erythrocyte sedimentation rate, C-reactive protein).

## Methods

### Study selection

Using the Preferred Reporting Items for Systematic Reviews and Meta-Analyses (PRISMA) guidelines, a systematic literature search was conducted [20]. Two independent reviewers screened article titles/abstracts and assessed the remaining full-text manuscripts for final inclusion. Reference lists of identified articles were also reviewed, and all relevant studies were included.

### Search strategy

A methodical search of the literature was performed using PubMed/MEDLINE, Scopus, and the Cochrane library from January 1, 1960, through January 23, 2021. The search strategy used the following keywords: ((Shoulder OR Glenohumeral*) AND (Sepsis OR Septic)). The search results were not initially filtered by language to identify both English and non-English studies that could be translated.

### Eligibility criteria

All studies with Level-I to IV evidence in the English/Spanish language were considered for inclusion. Other inclusion criteria included (1) studies on septic arthritis of the native shoulder joint that reported on at least one of the following parameters: joint aspiration data (i.e., synovial white cell count, gram stain, and culture results), preoperative serum markers (i.e., white blood cell count (WBC), erythrocyte sedimentation rate (ESR), C-reactive protein (CRP)), blood culture, presenting symptoms, patient comorbidites, and (2) studies involving multiple joints (i.e., pooled data) in which the data of interest (i.e., shoulder joint) could be isolated and extracted. Articles were excluded if they were (1) not transcribed in English/Spanish, (2) published before January 1, 1960, (3) skeletally immature patients (< 18 years of age), (4) studies reporting on postoperative shoulder infection (i.e., prosthetic joint infection or following mini-open/arthroscopic procedures), (5) studies reporting on periarticular shoulder sepsis (i.e., not involving the glenohumeral joint), and (6) book chapters, review articles, or opinion papers. A native shoulder was defined as any shoulder that had not undergone previous surgical intervention before the development of septic arthritis.

### Data abstraction and quality analysis

Two independent and blinded reviewers collected study data. Extracted data included: publication year, study design, level of evidence, sample size, age, sex, follow-up duration, clinical findings, imaging findings, laboratory values, time to presentation, preoperative and operative procedures, revisions, and comorbidities.

Study quality was evaluated by two independent investigators using the Methodological Index for Non-Randomized Studies (MINORS) criteria [21]. Each of the 12 items was graded from zero to two. The maximum cumulative scores were 24 for comparative studies and 16 for noncomparative studies.

### Outcome measures

The primary outcome of interest was the synovial white cell count from infected native shoulders. Secondary outcome measures include reporting on all lab values that may influence the diagnosis of shoulder sepsis.

### Statistical analysis

Due to the heterogeneity in how studies presented diagnostic methods or treatment outcomes, the data obtained from the selected studies were not adequate to perform a meta-analysis. For these reasons, a descriptive approach to data analysis was performed. Descriptive statistics, including means, proportions, ranges, and the intraclass correlation coefficient (ICC) were calculated using Stata software (v16.0, Stata Corp, College Station, Texas, USA, 2019).

## Results

### Search results

**Fig. 1:**
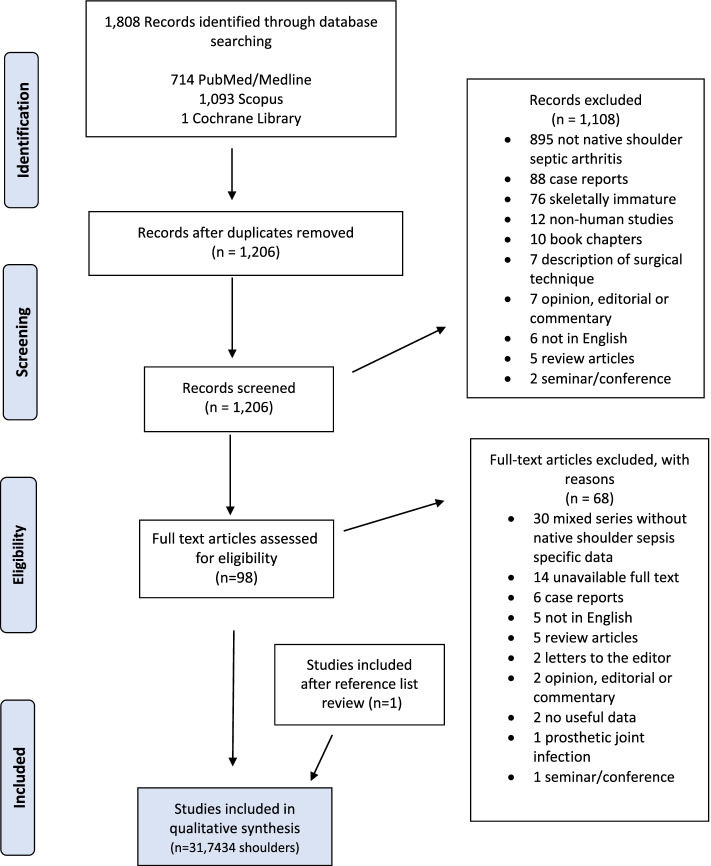
PRISMA flow diagram

The initial literature search yielded a total of 1808 studies. After duplicate removal, 1206 studies underwent title and abstract screening. Using our eligibility criteria, 1108 manuscripts were excluded, leaving 98 articles for full-text review. Following full-text review, 68 articles were removed, and one article was added after reviewing the reference lists of included studies, resulting in 31 studies [1–3, 5, 6, 8–10, 12–14, 16–19, 22–37] for final analysis (Fig. 1).

**Fig. 2:**
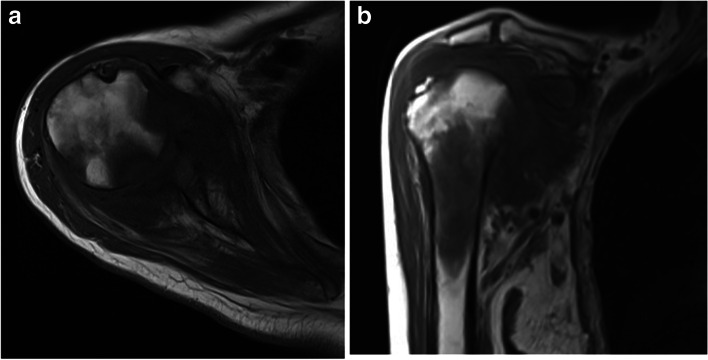
Axial (**A**) and coronal (**B**) MR imaging of a right shoulder in a patient with delayed diagnosis and treatment of shoulder sepsis. Findings reveal osteomyelitis of both the humeral head and glenoid vault and secondary arthritis

### Study characteristics

In total, there were 25 retrospective case series, five retrospective cohort studies and one retrospective case-control study included. There was a total of 7419 patients (7434 shoulder joints). Twenty-seven studies reported patient sex, which consisted of 4238 male and 2830 female patients. There were 17 studies (2 comparative/15 noncomparative) that reported on follow-up duration (range, 1 to 103.3 months) (Table 1).

**Table 1:** Study characteristics and demographic data

Study	Year	Study Design	LOE	Mean MINORS Score	Mean FU, mo	Patients/Shoulders, n	Mean Age, yr	Male/Female, n
Armbuster et al. [22]	1977	Case series	IV	6	NR	5/5	63.4	5/0
Master et al. [12]	1977	Case series	IV	5	NR	7/8	63	7/0
Gelberman et al. [1]	1980	Case series	IV	7	> 6	15/16	58	NR
Leslie et al. [6]	1989	Case series	IV	8	31.2	18/18	63	14/4
Pfeiffenberg et al. [23]	1996	Case series	IV	9	NR	14/14	57	NR
Lossos et al. [5]	1998	Case series	IV	9	NR	6/6	76	4/2
Wick et al. [24]	2003	Case series	IV	9	NR	11/11	52.1	NR
Chanet et al. [25]	2005	Case series	IV	8	NR	6/6	67.6	0/6
Smith et al. [9]	2005	Case series	IV	7	54	17/20	64	11/6
Jeon et al. [2]	2006	Case series	IV	9	16.4	19/19	59 (23–89)	17/2
Duncan et al. [8]	2008	Case series	IV	10	6	19/19	75.5 (49–94)	NR
Rhee et al. [26]	2008	Case series	IV	9	30	13/13	56	10/3
Kirchhoff et al. [10]	2008	Case series	IV	12	NR	25/25	66.5	15/10
Klinger et al. [3]	2010	Case series	IV	11	35.4	21/23	64.7	10/11
Matsuhashi et al. [27]	2011	Case series	IV	9	103.3	10/10	61.7	4/6
Abdel et al. [28]	2013	Case series	IV	10	31	46/50	66	35/11
Garofalo et al. [29]	2014	Case series	IV	8	NR	10/10	67.9	8/2
Cho et al. [30]	2016	Case series	IV	12	32.4	32/34	61.8	15/17
Jung et al. [31]	2016	Case series	IV	12	14	68/68	66.4	39/29
Sobreira et al. [32]	2016	Case series	IV	7	12.2	7/8	74	4/3
Böhler et al. [33]	2017	Retrospective cohort	III	20	NR	59/59	72	25/34
Jiang et al. [17]	2017	Retrospective cohort	III	16	NR	Group 1: 1223/1223 Group 2: 4355/4355 Group 3: 799/799	Group1; 62.8 Group 2: 60.6 Group3: 62	Group 1:682/541 Group 2: 2661/1694 Group 3: 490/309
Kim et al. [34]	2018	Retrospective cohort	III	21	Group s: 32.9 Group r: 30.8	Group s: 29/29 Group r:13/13	Group s: 61 Group r: 67.5	Group s:14/15 Group r:7/6
Sweet et al. [35]	2018	Case series	IV	11	83.1	97/97	58.2	58/39
Gramlich et al. [16]	2019	Case series	IV	10	NR	29/29	73 (38–93)	19/10
Lee et al. [13]	2019	Retrospective cohort	III	18	Group 1: 28.8 Group 2: 28.8	Group 1: 27/27 Group 2: 30/30	Group 1: 54.9 Group 2: 56.3	Group 1: 12/15 Group 2: 19/11
Joo et al. [18]	2020	Retrospective case-control	III	20	NR	97/97	61	53/44
Khazi et al. [19]	2020	Retrospective cohort	III	16	1	204/204	NR	133/71
Kwon et al. [36]	2020	Case series	IV	10	14.3	35/36	63.8	15/20
Rhee et al. [14]	2020	Case series	IV	14	27.6	31/31	54.9	11/20
Takahasi et al. [37]	2020	Case series	IV	11	NR	22/22	67.9	10/12

*Abbreviations*: *LOE* Level of Evidence, *FU* Follow-up, *NR* Not reported

Note for studies consisting of more than one group, average age shows each group’s average

### Study quality

Overall, the average MINORS score was nine for the non-comparative studies and 15 for the comparative studies (Table 1). Of the 31 studies, 25 were level IV and six level III evidence. The 25 level IV evidence studies lacked unbiased assessment or reporting of appropriate study endpoints. The overall inter-rater agreement (ICC) for the MINORS score between the two investigators was 0.98 (95% CI, 0.88–0.99).

### Clinical presentation

Of the 31 included studies, only seven studies (138 of 7434 shoulders, 1.9%) quantified presenting symptoms: 128 patients experienced pain (93%), 101 reported swelling (73%), 59 reported limited range of motion (43%), and 39 reported redness (28%). Other symptoms reported included general fatigue and malaise. In nine of the studies (29%), the average duration between symptom onset and clinical presentation/diagnosis was reported and ranged from 4.3 days to 150 days (Table 2).

**Table 2:** Symptom duration and hospitalization data

Study	Symptom Onset to Presentation, days	Time from Presentation to Surgery, days	Reoperation Rate, %	Average Hospitalization, days
Armbuster et al.	NR	NR	NR	NR
Master et al.	NR	NR	NR	NR
Gelberman et al.	NR	NR	NR	NR
Leslie et al.	NR	NR	NR	NR
Pfeiffenberg et al.	24	NR	42%	NR
Lossos et al.	4.3	NR	NR	28.83
Wick et al.	NR	NR	NR	NR
Chanet et al.	75.8	NR	NR	NR
Smith et al.	NR	NR	NR	NR
Jeon et al.	NR	21	26%	NR
Duncan et al.	NR	NR	26%	NR
Rhee et al.	21	13	NR	NR
Kirchhoff et al.	14.6	NR	NR	26.9
Klinger et al.	16 (5–76)	NR	NR	NR
Matsuhashi et al.	NR	18.6	NR	NR
Abdel et al.	8 (1–60)	3 (0–15)	32%	NR
Garofalo et al.	75–150	NR	0%	24 (17–32)
Cho et al.	NR	23.3	14.7%	NR
Jung et al.	NR	17.5	1%	NR
Sobreira et al.	NR	42	13%	NR
Böhler et al.	NR	NR	30.5%	12
Jiang et al.	NR	NR	12.30%	NR
Kim et al.	NR	Group s: 8.9 Group r: 8.1	31%	Group s: 25.4 Group r: 39.7
Sweet et al.	8.2 (1–35)	NR	35%	NR
Gramlich et al.	NR	NR	NR	83%
Lee et al.	Group 1: NR Group 2: NR	Group 1: NR Group 2: NR	Group 1: 30% Group 2: 8%	Group 1: NR Group 2: NR
Joo et al.	NR	NR	NR	NR
Khazi et al.	NR	NR	Arthroscopy: 10.2% Open: 15.79%	NR
Kwon et al.	NR	10.9	5.6%	NR
Rhee et al.	NR	NR	54.8%	NR
Takahasi et al.	NR	NR	14%	NR

*Abbreviations*: *NR* Not reported, *MRI* Magnetic Resonance Imaging, *CT* Computed Tomography

### Laboratory findings

Only 20 studies (709 of 7434 shoulders, 9.5%) reported on the average preoperative serum WBC counts (average values ranged from 9390 cells/mcL to 15,500 cells/mcL). Of these 709 shoulders, 402 shoulders (57%) had elevated WBC (i.e., greater than 11,000 cells/mcL). Elevated average ESR values (i.e., greater than 20 mm/h) were found in all 18 studies (641 shoulders, 8.6%) reporting ESR (100% positivity rate), which ranged from 41.5 mm/h to 120 mm/h. Of the 16 studies (660 shoulders, 8.9%) that reported average CRP levels, only 313 shoulders (47%) had an elevated average CRP (i.e., greater than 10.0 mg/L) with a range of 4.7 mg/L to 134 mg/L. Ten studies (385 shoulders, 5.2%) documented average synovial white cell counts (average values ranged from > 30,000 cells/mm^3^ to 195,667 cells/mm^3^). Of these studies, nine (90%) reported a high (i.e., greater than 50,000 cells/mm^3^) average synovial cell count. Twenty-nine of the 31 studies (853 shoulders, 11.5%) reported synovial aspiration culture results: 74% were positive, and 26% were negative. Gram stains were only reported in four studies (93 results): 39% were positive and 61% were negative. Administration of antibiotics prior to joint aspiration was inconsistently reported for the majority of studies. For the 18 studies reporting either a negative culture or gram stain, 6 of the studies (33%) reported antitibiotic administration before aspiration and five studies (28%) did not report when they administered antibiotics. Of the 29 studies (4 comparative/ 25 non-comparative) that reported the causative organisms, 404 of the 853 shoulder joints (47%) involved *Staphylococcus aureus* (including methicillin-sensitive and methicillin-resistant) (Table 3).

**Table 3:** Laboratory findings

Study	WBC, cells/mcL	ESR, mm/h	CRP, mg/L	Pre-operative Aspiration	Aspiration – Gram Stain & Culture	Aspiration – Cell Count, (% PMN)	Organisms, (n)
Armbuster et al.	NR	NR	NR	Yes: 5	Culture positive: 5	195,667 (NR)	*S. aureus* (2) *E. coli* (1) D. pneumoniae (1) *A. hydrophila* (1)
Master et al.	NR	NR	NR	Yes: 8	Culture positive: 8	146,416 (NR)	*S. aureus* (4) *S. pneumoniae* (1) *E. coli* (1) *A. hydrophila* (1)
Gelberman et al.	NR	NR	NR	Yes: 15 No:1	Culture positive: 16	100,678 (NR)	*S. aureus* (7) Group B S. (3) Aeromonas (2) *E. coli* (1) *S. pneumoniae* (1) Alpha Strep. (1) Coagulase-negative Staph. (1)
Leslie et al.	5500–14,100 (16)	25–120 (11)	NR	Yes: 18	Culture positive: 17 Culture negative: 1	NR	*S. aureus* (11) *E. coli* (3) *S. pneumoniae* (1) *S. viridans* (1) *P. mirabilis* (1) M tuberculosis (1)
Pfeiffenberg et al.	11,860 (5/14)	62.4 (11/14)	NR	No: 14 (intraop)	Culture positive: 14	NR	*S. aureus* (9) *S. epidermidis* (3), Gram positive cocci (2), Gram negative cocci (1), *S. haemolyticus* (1), M tuberculosis (1)
Lossos et al.	11,050	95.2	NR	Yes: 6	Gram stain positive:1 Gram stain negative: 5 Culture positive: 6	NR	*S. aureus* (4) *P. aeruginosa* (1) *E. coli* (1)
Wick et al.	NR	NR	4.67	Yes:11	Culture positive:11	NR	*S. aureus* (11)
Chanet et al.	9670	99.8	NR	Yes: 6	Culture positive: 6	NR	*S. aureus* (5) Polymicrobial (1)
Smith et al.	15,500	69	NR	Yes: 20	Gram stain positive: 11 Gram stain negative: 9 Culture positive: 17 Culture negative:3	114,000 (NR)	*S. aureus* (14) Polymicrobial (2) Gram positive bacillus (1)
Jeon et al.	NR	NR	NR	Yes: 19	Culture positive:13 Culture negative: 6	NR	MSSA (7) *P. aeruginosa* (3) Pneumococcus (1) Acinetobacter (1) *E. coli* (1)
Duncan et al.	10,500	66	NR	Yes:19	Gram stain positive: 16 Gram stain negative: 3 Culture positive: 17 Culture negative: 2	NR	MSSA (5) MRSA (1) Group B S. (5) *S. epidermidis* (3) *S. viridans* (1) *E. coli* (1) P. bacterium (1)
Rhee et al.	12,700	41.5	9.5	Yes: 13	Culture positive: 10 Culture negative: 3	NR	MSSA (6) MRSA (4)
Kirchhoff et al.	NR	NR	NR	Yes:25	Culture positive: 23 Culture negative: 2	> 30,000 (NR)	*S. aureus* (21) *S. pneumoniae* (1) *E. coli* (1)
Klinger et al.	NR	67 (45–142)	134 (76–213)	Yes: 23	Culture positive: 20 Culture negative: 3	NR	*S. aureus* (17) MRSA (2) *S. epidermidis* (3)
Matsuhashi et al.	12,666	NR	14.9	Yes: 10	Culture positive: 10	NR	*S. aureus* (6) *S. epidermidis* (4)
Abdel et al.	13,000	66	83	Yes: 45 No: 5	Gram stain positive: 8 Gram stain negative: 42 Culture positive: 41 Culture negative:9	110,988 (87%)	MSSA (22) MRSA (10) Group B Streptococcus (9) *S. viridans* (1) *S. epidermidis* (1) Group C Streptococcus (1) Haemophilus (1) Pneumonococcus (1)
Garofalo et al.	12,000–14,000 (3/10)	58 (38–86)	128 (84–144)	Yes: 3 No: 7	Culture positive: 6 Culture negative: 4	NR	Coagulase-negative Staph. (2) *S. epidermidis* (2) *P. aeruginosa* (1) *P. acnes* (1)
Cho et al.	10,400	59.2	9.5	Yes: 34	Culture positive: 13 Culture negative: 21	NR	MSSA (7) MRSA (2) *S. epidermidis* (1) *S. equi* (1) Providencia (1) Burkholeria (1) *P. aeruginosa* (1) Candida (1)
Jung et al.	10,263	78.1	8.46	Yes: 68	Culture positive: 43 Culture negative: 25	NR	MSSA (16) MRSA (9) Streptococcus spp. (6) *P. aeruginosa* (4) Enterococcus (4) Other pathogens (4)
Sobreira et al.	NR	NR	NR	Yes: 8	Culture positive: 5 Culture negative: 3	NR	*S. aureus* (4) *E. coli* (1)
Böhler et al.	11,300	NR	14.3	Yes: 59	Culture positive: 31 Culture negative: 28	NR	MSSA (18) MRSA (4) Streptococcus spp. (4) Other Staphylococcus spp. including S epidermidis (5) *P. aeruginosa* (2) Enterobacter (1)
Jiang et al.	NR	NR	NR	NR	NR	NR	MSSA 39% MRSA 21% Streptococcus 11% Gram negative 7% (in subgroup)
Kim et al.	Group s: 10,209 Group r: 12,200	Group s: 81.1 (0–28) Group r: 92.8	Group s: 129.3 Group r: 219.9	Yes: 42	Culture positive: 22 Culture negative: 20	NR	MRSA (6) MSSA (6) *P. aeruginosa* (3) *S. marcescens* (1) Streptococcus-unspecified (4) Enterobacter (2)
Sweet et al.	13,400	81.9	15.9	NR	Culture positive: 97	121,656 (92.6)	MSSA 37.1%, MRSA 25.7%, *S. pneumoniae* 5%, Group B Streptococcus 5%, Pseudomonas 5%
Gramlich et al.	NR	NR	NR	Yes: 29	Culture positive: 29	NR	*S. aureus* 47% *S. epidermidis* 27% *P. acnes* 13% Enterococcus spp. 3% Streptococcus spp. 3% *M. morganii* 3% *F. magna* 3%
Lee et al.	Group 1: 12,380 Group2: 11,540	Group1:63.2 Group2:59.2	Group1: 7.86 Group2: 7.67	Group1: Yes: 27 Group2: Yes: 30	Group 1 Culture positive: 20 Culture negative: 7 Group 2 Culture positive: 22 Culture negative: 8	Group1: NR Group2: NR	Group 1: MSSA (8) MRSA (2) *S. epidermidis* (6) Pseudomonas (2) Corynebacterium (2) Group 2: MSSA(10) MRSA (3) S epidermidis (6) Pseudomonas (2) Corynebacterium (1)
Joo et al.	10,110	70	7.4	Yes: 97	Culture positive: 36 Culture negative: 61	100,600 (NR)	MRSA (22) MSSA (2) *S. epidermidis* (5) *E. coli* (2) S. agalactieae (2) S. sanguis (1) Acinetobacter (1) Klebsiella (1)
Khazi et al.	NR	NR	NR	NR	NR	NR	NR
Kwon et al.	9390	60.3	9.23	Yes: 36	Culture positive: 15 Culture negative: 21	128,867 (88.30)	MSSA (6) MRSA (4) *S. epidermidis* (1) Coagulase-negative Staphylococcus (1) *S. pneumoniae* (1) Serratia (1) Group B Streptococcus (1)
Rhee et al.	12,600	63.5	7.54	Yes: 29 No:2	Culture positive: 24 Culture negative: 7	98,911 (NR)	MSSA (10) MRSA (4) *S. epidermidis* (6) MRSA *P. aeruginosa* (2) Corynebacterium (2)
Takahasi et al.	Group 1: 11,590 Group 2: 9550	NR	Group1: 15.1 Group 2: 8.7	Yes: 22	Culture positive: 19 Culture negative: 3	NR	MSSA (10) MRSA (4) Streptococcus spp. (4) *P. aeruginosa* (1)

*Abbreviations*: *NR* Not reported, *WBC* White blood cell count, *ESR* Erythrocyte sedimentation rate, *CRP* C-reactive protein, *MSSA* Methicillin-Sensitive *Staphylococcus aureus*, *MRSA* Methicillin-Resistant *Staphylococcus aureus*; Intraop, intraoperative

## Discussion

The diagnosis of shoulder sepsis remains undefined despite the abundance of literature on the subject. Left untreated or diagnosed late, shoulder sepsis can lead to irreversible chondral, osseous, and soft-tissue damage, patient morbidity, and even death [1–3, 5]. Septic arthritis of the shoulder has also been associated with a reoperation rate as high as 30%, further increasing the risk of perioperative complications and patient morbidity [38]. This systematic review emphasizes the need to modify our understanding of native shoulder sepsis presentation and diagnosis. Due to its relative rarity compared with other joints, there is a paucity of uniform data reporting its diagnosis. Applying the principles of knee septic arthritis evaluation to the shoulder may not produce the same results. This systematic review identified some differences and other similarities in the traditional diagnosis of septic arthritis. Namely, the aspiration values seem unique to shoulder sepsis as the joint capsule is prone to failure with spread of infection to other periarticular zones resulting in decreased pain and diagnostic delay. In this setting aspiration values are less specific.

Of the three reported serum laboratory findings, the most commonly reported value was the serum WBC count. This study demonstrates that not all patients with shoulder sepsis have elevations in their serum WBC (57%) and CRP (47%). For example, Leslie et al. [6] reported on six patients (33%) and Garofalo et al. [29] on seven patients (70%) with a normal serum WBC at the time of diagnosis. In the study by Pfeiffenberger et al., [23] only five out of 14 patients (36%) had an elevated serum WBC, averaging 11,860 cells/mcL. These findings question the diagnostic utility of serum WBC and CRP for shoulder sepsis, which compares favourably with the literature. Li et al. [39] examined these lab markers and found serum WBC and ESR to be poor tests, whereas synovial WBC was the best diagnostic tool for septic arthritis of all joints. Though this study was limited by its small sample size, Margaretten et al. [40] solidified these findings in their comprehensive meta-analysis on septic arthritis involving all peripheral joints. They confirmed that the two most powerful tools were the synovial WBC and percentage of polymorphonuclear cells from arthrocentesis, the latter being reported in only three (9.7%) of the studies in this review.

One of the most interesting findings in this systematic review was the reported synovial white cell counts in patients with shoulder sepsis. Although the synovial white cell count was high (> 50,000 cells/mm^3^) in 90% of studies reporting such data, this represented only 370 of the 7434 shoulders (5.0%) included in this review. In their series of 43 patients with native shoulder sepsis, Kirchhoff et al. [10] reported how glenohumeral joint sepsis could occur in patients with a relatively lower synovial white cell count (> 30,000 cells/mm^3^ in all their joint aspirates). Notably, the majority of their patients were diagnosed at 14.6 days of symptom onset, which could explain the discrepancy in reported aspirated cell counts. Abdel et al. [28] and Sweet et al. [35] were the only other studies that reported on synovial white cell counts and the temporal sequence between symptom onset and presentation. Both of these studies reported an average synovial white cell count over 110,000 cells/mm^3^, with an average time to presentation of 8 days. Therefore, time to diagnosis may influence the aspirated WBC count, where longer times to presentation may mitigate the body’s inflammatory/immune response, which is subsequently reflected by lower aspiration cell count values. With time, ongoing infection may compromise the integrity of the shoulder capsule, allowing the infection to spread to other areas about the shoulder girdle that manifest with lower synovial white cell counts. Of note, these differences in synovial cell counts may also be explained by the temporal relationship of antibiotic administration and synovial fluid aspiration [41]. Though the timing of antibiotic administration is inconsistently reported, all of the patients in Abdel et al.’s [28] case series received antibiotics after aspiration where as all of the patients in Kirchhoff et al.’s [10] series were given antibiotics before aspiration, which could have mitigated the number of cells aspirated. Furthermore, most of the included studies in this review excluded patients with osteomyelitis, which could have biased results towards a much earlier presentation of shoulder sepsis that may have a stronger inflammatory/immune response, yielding higher synovial white cell counts. This is an important consideration when the diagnostic threshold for typical septic arthritis in other joints is an aspirated cell count greater than 50,000 cells/mm^3^ [39, 40]. Collectively, the lack of studies reporting on aspiration cell counts demonstrates inconsistencies in utilizing a laboratory value that is conventionally diagnostic of septic arthritis in other joints.

The presented systematic review has both strengths and limitations. We believe our study effectively evaluates contemporary diagnostic measures taken to manage septic arthritis of the shoulder. To the best of the authors’ knowledge, no other systematic review has analysed the methods in which shoulder sepsis may differ from other joints, thereby necessitating a separate diagnostic and management protocol. However, this review is primarily limited by the diversity of diagnostic data and outcome reporting (i.e., less than 10% reporting of primary and secondary data) specific to native shoulder sepsis. To date, there is no standardized approach to shoulder sepsis, so many studies lack uniformity, resulting in inconsistent documentation of serum markers, and arthrocentesis findings. Additionally, most of the included studies were retrospective (level III or IV evidence), introducing inherent bias associated with the data retrieval process. Therefore, we are unable to provide definitive recommendations on the diagnostic workup of shoulder sepsis, and our conclusions remain limited.

## Conclusion

This systematic review underscores the need to modify our understanding of the evaluation and diagnosis of septic arthritis of the shoulder. Shoulder sepsis presentation differs from other joints in substantial ways, and this warrants a separate and tailored approach. Aspiration results and serum markers may be related to the time interval between symptom onset and diagnosis. Patients may present with normal serum WBC and CRP levels and conventionally lower synovial WBC. This study does not suggest that synovial fluid aspiration of the shoulder is of low value when done in the acute setting. Synovial cell counts are underutilized and implementing this diagnostic test in the acute setting could help prevent underdiagnosis and subsequent undertreatment of patients with native shoulder joint sepsis.

## Data Availability

The authors declare that the data supporting the findings of this study are available within the article. The data that support the findings of this study is available in Pubmed/Medline.

## Abbreviations

MRI: Magnetic Resonance Imaging
PRISMA: Preferred Reporting Items for Systematic Reviews and Meta-Analyses
WBC: White blood cell count
ESR: Erythrocyte sedimentation rate
CRP: C-reactive protein
MINORS: Methodological Index for Non-Randomized Studies
ICC: Intraclass correlation coefficient
